# Evaluation of a weighting approach for performing sensitivity analysis after multiple imputation

**DOI:** 10.1186/s12874-015-0074-2

**Published:** 2015-10-13

**Authors:** Panteha Hayati Rezvan, Ian R. White, Katherine J. Lee, John B. Carlin, Julie A. Simpson

**Affiliations:** Centre for Epidemiology and Biostatistics, Melbourne School of Population and Global Health, The University of Melbourne, Parkville, Melbourne, VIC Australia; MRC Biostatistics Unit, Cambridge Institute of Public Health, Cambridge, CB2 0SR UK; Clinical Epidemiology and Biostatistics Unit, Murdoch Childrens Research Institute, Parkville, Melbourne, VIC Australia; Department of Paediatrics, The University of Melbourne, Parkville, Melbourne, VIC Australia

**Keywords:** Multiple imputation, Selection model, Missing not at random, Weighting approach, Sensitivity analysis

## Abstract

**Background:**

Multiple imputation (MI) is a well-recognised statistical technique for handling missing data. As usually implemented in standard statistical software, MI assumes that data are ‘Missing at random’ (MAR); an assumption that in many settings is implausible. It is not possible to distinguish whether data are MAR or ‘Missing not at random’ (MNAR) using the observed data, so it is desirable to discover the impact of departures from the MAR assumption on the MI results by conducting sensitivity analyses. A weighting approach based on a selection model has been proposed for performing MNAR analyses to assess the robustness of results obtained under standard MI to departures from MAR.

**Methods:**

In this article, we use simulation to evaluate the weighting approach as a method for exploring possible departures from MAR, with missingness in a single variable, where the parameters of interest are the marginal mean (and probability) of a partially observed outcome variable and a measure of association between the outcome and a fully observed exposure. The simulation studies compare the weighting-based MNAR estimates for various numbers of imputations in small and large samples, for moderate to large magnitudes of departure from MAR, where the degree of departure from MAR was assumed known. Further, we evaluated a proposed graphical method, which uses the dataset with missing data, for obtaining a plausible range of values for the parameter that quantifies the magnitude of departure from MAR.

**Results:**

Our simulation studies confirm that the weighting approach outperformed the MAR approach, but it still suffered from bias. In particular, our findings demonstrate that the weighting approach provides biased parameter estimates, even when a large number of imputations is performed. In the examples presented, the graphical approach for selecting a range of values for the possible departures from MAR did not capture the true parameter value of departure used in generating the data.

**Conclusions:**

Overall, the weighting approach is not recommended for sensitivity analyses following MI, and further research is required to develop more appropriate methods to perform such sensitivity analyses.

**Electronic supplementary material:**

The online version of this article (doi:10.1186/s12874-015-0074-2) contains supplementary material, which is available to authorized users.

## Background

The problem of missing data is frequently encountered in clinical and epidemiological research, in particular, in longitudinal cohorts with multiple waves of data collection [1–5]. Excluding individuals with missing data from the statistical analysis (i.e. complete case analysis (CC)) can lead to biased inference since individuals with complete records do not typically represent the study population under investigation [6, 7].

Several statistical techniques have been developed in recent decades to address the issue of missing data [8]. Multiple imputation (MI), which is widely available in standard software packages (e.g. R [9], SAS [10] and Stata [11]), is one of the most flexible approaches for handling missing data [12–14].

MI begins by replacing the missing data with plausible values by sampling multiple times from an imputation model; thus, multiple completed (observed plus imputed) datasets are created. Each completed dataset is then analysed separately using standard statistical methods, and the resulting point and interval estimates are combined using Rubin’s rules to obtain an overall MI inference for the parameter(s) of interest [7, 15, 16].

The validity of estimates obtained from MI rests on a key assumption concerning the mechanism underlying the missing data. As usually implemented in standard statistical software, MI assumes that data are ‘Missing At Random’ (MAR), i.e. that the probability of missingness does not depend on the missing data after conditioning on the observed data. Contrary to this MAR assumption it is often plausible in practice that differences in the data distribution between individuals with missing items and those with complete data cannot be explained by the observed data alone, in which case the data are ‘Missing Not At Random’ (MNAR). Performing MI under MAR when the actual missingness process is MNAR may produce biased estimates [17].

Unfortunately, distinguishing between MAR and MNAR data is not possible using the observed data as by definition the reasons for missing data under MNAR are not observed. Consequently, researchers have suggested approaches to investigate the sensitivity of the MI results to departures from the MAR assumption. Two approaches that can be implemented within the MI framework have been proposed: a weighting approach based on a selection model [6, 18] and a pattern-mixture approach [6, 19–21]. These methods are based on the two general approaches to factorising the joint distribution of the response and missing data mechanism associated with the response: the selection model and the pattern-mixture model [7, 22–26]. At present, most available software packages do not include features for conducting sensitivity analysis using the weighting approach within the suite of commands that are available for performing MI. However, SAS (SAS/STAT 13.1 [27]) and R (SensMice package [28]) have recently introduced the pattern-mixture approach for performing sensitivity analyses to the MAR assumption.

The weighting approach is a specific application of the selection model that has been developed within the MI framework to assess the robustness of conclusions to an assumed MNAR mechanism. This approach is an approximate and fast computational method for performing a ‘local’ sensitivity analysis [29] after implementing MI, and typically deals with problems in which there is a single variable with missing data [18]. Using the weighting approach, the estimates obtained under the MAR assumption from a standard MI procedure are re-weighted in such a way that they reflect the MNAR mechanism.

In this paper we comprehensively evaluate the weighting approach for performing a sensitivity analysis after implementing the standard MI procedure under the MAR assumption, and describe possible problems that might arise from applying this approach. We assess the proposed approach by estimating the marginal mean of a partially observed variable and a measure of association between the partially observed variable and a completely observed variable, across different numbers of imputations and sample sizes, and where the degree of departure from MAR vary from moderate to large.

The structure of this paper is as follows. We begin with an overview of selection models and multiple imputation. This is followed by an explanation of the weighting approach and the theory behind this method (which is based on importance sampling). We also describe the graphical diagnostics proposed by Carpenter et al. [18] for exploring the first condition of importance sampling and then apply it to a single simulated dataset. We evaluate the performance of the weighting approach using simulation studies in which we investigate whether the method provides unbiased estimates of the parameter of interest. Then, we discuss why the application of the method of importance sampling in the weighting approach might go wrong. We address the question of how to choose the sensitivity parameter (i.e. a parameter representing the extent of departure from MAR) and describe a graphical method proposed by Héraud-Bousquet et al. [30]. We critique the graphical method using the single simulated dataset presented earlier and show that there is no alternative to using subject-matter knowledge. Finally, we conclude with a discussion of the weighting approach and its limitations.

### Selection models

As mentioned earlier, the basis of the weighting approach is a selection model. Before describing the weighting approach, we give a brief description of selection models.

In order to draw inference about missing data when the underlying missingness mechanism is MNAR, we need a joint model for the complete data and the missing data mechanism [6, 29]. Let *Y* be a partially observed outcome variable, *X* be a fully observed covariate and *R* be a missing value indicator, where *R* =1 if *Y* is observed and *R* = 0 otherwise. Then, the joint distribution of the complete data and the missing data mechanism can be written as

1 $$ f\left(Y,R\Big|X\right)=f\left({Y}_{obs},{Y}_{mis},R\Big|X\right) $$

where *Y*_*obs*_ and *Y*_*mis*_ represent the observed and missing components of the outcome variable*,* respectively. The joint distribution (1) can be represented as

2 $$ f\left({Y}_{obs},{Y}_{mis},R\left|X\right.\right)=f\left(R\left|{Y}_{obs},{Y}_{mis},X\right.\right)f\left({Y}_{obs},{Y}_{mis}\left|X\right.\right) $$

which factorises the distribution of the complete data and the missing data mechanism into a distribution of the missing data mechanism (*R*) conditional on the observed (*Y*_*obs*_) and missing data (*Y*_*mis*_), and the (marginal) distribution of the complete data. This factorisation of the joint distribution is known in the literature as the *selection model*. In general, selection modelling requires strong identifying assumptions because the data do not contain information (since *Y*_*mis*_ is not observed) about the required conditional distribution of *R*. Additionally, fitting these types of models requires complex computational algorithms and specific software for implementation [31–33]. Here we consider a selection model where the missing data mechanism is dependent on the fully observed *X* and partially observed *Y*:

3 $$ logit\left[ Pr\left(R=1\Big|X,Y\right)\right]=f(X)+\delta Y $$

In Equation (3), *δ* represents the change in the log-odds of *R* = 1 (i.e. of observing *Y*) for a one-unit change in *Y* holding *X* fixed, so this parameter represents the extent of departure from the MAR assumption. Equivalently, exp (*δ*) represents the relative change in the odds of observing *Y*. Note that in general estimating *δ* from the observed data is not possible since values of *Y* are not observed when *R* = 0 [34].

### Multiple imputation

We briefly describe the MI procedure for the partially observed outcome variable *Y* and the fully observed covariate *X* defined in the previous section.

MI proceeds with replacing the values of the missing data *Y*_*mis*_ by multiple (*m*) values drawn from the posterior predictive distribution of the missing data *f*(*Y*_*mis*_|*Y*_*obs*_, *X*). The standard analysis is then carried out for each of the *m* completed datasets (observed plus imputed), which results in *m* sets of parameter estimates $\left({\widehat{\theta}}_j\right)$ and associated estimated variances ((*s. e*. (*θ*_*j*_))^2^). A combined estimate of the parameter of interest $\left({\widehat{\theta}}^{MAR}\right)$, along with its variance $\left(V\left({\widehat{\theta}}^{MAR}\right)\right)$ is then obtained using Rubin’s rules. The standard MI estimate is given by:

4 $$ {\widehat{\theta}}^{MAR}=\frac{1}{m}{\displaystyle {\sum}_{j=1}^m{\widehat{\theta}}_j} $$

where *m* is the number of imputations and ${\widehat{\theta}}_j$ is the parameter estimate for the analysis of interest (which hereafter will be termed the ‘target analysis’) obtained from the *j*^*th*^ imputed dataset. The estimated variance of the standard MI estimate $\left(V\left({\widehat{\theta}}^{MAR}\right)\right)$ allows for between–and within–imputation variability:

5 $$ V\left({\widehat{\theta}}^{MAR}\right)={V}_W\left({\widehat{\theta}}^{MAR}\right)+\left(1+\frac{1}{m}\right)\times {V}_B\left({\widehat{\theta}}^{MAR}\right) $$

where the estimated within-imputation variance is ${V}_W\left({\widehat{\theta}}^{MAR}\right)=\frac{1}{m}{{\displaystyle {\sum}_{j=1}^m\left(s.e.\left({\theta}_j\right)\right)}}^2$ and the estimated between-imputation variance is ${V}_B\left({\widehat{\theta}}^{MAR}\right)=\frac{1}{m-1}{{\displaystyle {\sum}_{j=1}^m\left({\widehat{\theta}}_j-{\widehat{\theta}}^{MAR}\right)}}^2$ [7].

### The weighting approach

In the weighting approach, estimates obtained from the imputed datasets generated under the MAR assumption, via the standard MI procedure, are re-weighted in order to provide an overall parameter estimate that would be valid if the data were a particular form of MNAR [18]. In this approach, the weights given to the parameter estimates from each of the imputed datasets $\left({\widehat{\theta}}_j\right)$ are calculated based on the assumed magnitude of departure from MAR (*δ*), which might be chosen by expert judgement based on content-matter knowledge [35–37]. Alternatively, a researcher can examine how an inference about the parameter of interest changes as *δ* varies over a plausible range of values. *δ* = 0 indicates that the missing data mechanism is MAR; as *δ* moves away from zero there is a greater departure from MAR, or in other words a larger degree of MNAR. The weights are calculated as follows:

6 $$ {\tilde{w}}_j\left(\delta \right)= exp\left(-\delta {\varSigma}_{i\in {I}_Y}{Y}_{ij}\right) $$

where *Y*_*ij*_ indicates the imputed value of *Y* in the completed dataset *j* for the *i*^*th*^ individual and *I*_*Y*_ is the set of individuals with *Y* missing. A single weight $\left({\tilde{w}}_j\right(\delta ))$ is calculated for the *j*^*th*^ imputed dataset according to the degree of departure from MAR (*δ*) and the sum of the imputed values in that dataset. In particular, when *δ* > 0, the imputed dataset(s) with the smallest sum of imputed values is up-weighted, and when *δ* < 0, the imputed dataset(s) with the largest sum of imputed values is down-weighted. These are then normalised as follows:

7 $$ {w}_j\left(\delta \right)=\frac{{\tilde{w}}_j\left(\delta \right)}{{\displaystyle {\sum}_{j=1}^m{\tilde{w}}_j\left(\delta \right)}} $$

Note that following Carpenter et al. [18], *Y* is assumed to be an outcome variable for ease of exposition. It is unclear how this method would extend to missingness in multiple variables, except for the case where only one of the variables is MNAR [18]. The MNAR estimate is then defined as:

8 $$ {\widehat{\theta}}^{MNAR}\left(\delta \right)={\displaystyle {\sum}_{j=1}^m{w}_j\left(\delta \right)}\times {\widehat{\theta}}_j $$

The estimated variance of ${\widehat{\theta}}^{MNAR}\left(\delta \right)$ is calculated, assuming weighted versions of the within- and between- imputation variances:

9 $$ V\left({\widehat{\theta}}^{MNAR}\left(\delta \right)\right)\approx {V}_W\left({\widehat{\theta}}^{MNAR}\left(\delta \right)\right)+\left(1+\frac{1}{m}\right)\times {V}_B\left({\widehat{\theta}}^{MNAR}\left(\delta \right)\right) $$

where $$ {V}_W\left({\widehat{\theta}}^{MNAR}\left(\delta \right)\right)={\displaystyle {\sum}_{j=1}^m{w}_j\left(\delta \right)}\times {\left(s.e.\left({\theta}_j\right)\right)}^2 $$ and $$ {V}_B\left({\widehat{\theta}}^{MNAR}\left(\delta \right)\right)={{\displaystyle {\sum}_{j=1}^m{w}_j\left(\delta \right)\left({\widehat{\theta}}_j-{\widehat{\theta}}^{MNAR}\left(\delta \right)\right)}}^2 $$ [6, 18].

### Importance sampling

The weighting approach is based on the method of importance sampling [38, 39]. In this section we briefly explain how the weighting approach, as defined in the previous section, is an application of importance sampling.

The general idea of importance sampling is to estimate a property of a distribution of interest (e.g. ‘*g*’) by weighting the observations from a similar alternative distribution (e.g. ‘*f*’). According to the principles of importance sampling we can draw samples from the ‘*f’* distribution to inform about ‘*g*’ if:

- *f* supports the distribution of *g*, i.e. the support of *f* (defined as the range on which *f* > 0) includes the support of *g*, and
- the ratio *g/f*, known as the importance ratio or importance weight, is bounded by a constant quantity.

In simple words, the latter condition indicates that the importance ratios should not be extremely large. However, in some situations, a large proportion of importance weights take small values and a few importance weights take very large values. In such cases, applying importance sampling may introduce bias. In the literature, it has been suggested to examine the histogram of the logarithms of the importance weights to explore problems regarding high importance weights [38].

This theory was applied within the MI framework [18], in which ‘*g*’ was identified with the imputation distribution under MNAR and ‘*f*’ with the imputation distribution under MAR. Returning to the example explained earlier, where *Y* is a variable with some values missing, with a missingness indicator *R,* which is 1 if *Y* is observed and 0 otherwise, and *X* is a fully observed variable, for this case, ‘*g*’ and ‘*f*’ correspond to *f*[*Y*|*X*, *R* = 0] (a desired distribution that we wish to draw from (i.e. impute under MNAR)) and *f*[*Y*|*X*, *R* = 1] (the imputation distribution under MAR), respectively. Carpenter et al. [18] claimed that, under a particular form of the logistic regression model of *R* on *X* and *Y* in Equation (3), the importance weight (i.e. the ratio $\frac{g}{f}=\frac{f\left[Y\left|X,R=0\right.\right]}{f\left[Y\left|X,R=1\right.\right]}$) for imputation *j* is ${\tilde{w}}_j\left(\delta \right)= exp\left(-\delta {\varSigma}_{i\in {I}_Y}{Y}_{ij}\right)$ (Equation (6)), which depends on the magnitude of departure from MAR (*δ*) and the sum of imputed values (*Y*_*i*_) in the imputation *j*.

These weights are used to re-weight the estimates obtained from each imputed dataset under MAR to provide an overall estimate under MNAR. Carpenter and Kenward [6] claimed that, within the MI framework, the two conditions of importance sampling described above equate to:

- the MNAR estimate $\left({\widehat{\theta}}^{MNAR}\left(\delta \right)\right)$ needs to be within the range of the MAR estimates from each of the imputed dataset $\left({\widehat{\theta}}_js\right)$ since the MNAR estimate is the re-weighted average of the MAR estimates (i.e. there is a shared support for the distribution of the parameter of interest under MNAR and MAR), and
- the ratio of the distribution of imputations under MNAR to the distribution of imputations under MAR must be bounded.

They argued that if the proposed conditions of importance sampling are satisfied, the accuracy of the estimate under the MNAR assumption and its associated variance will improve with increasing the number of imputations (*m* → ∞). According to their suggestion the number of imputations should be large (*m* ≥ 50) when using the weighting approach following MI. They concluded that this approach is suitable for performing a local sensitivity analysis after MI under MAR to account for missing data that are weakly MNAR.

### Graphical diagnostics

Carpenter et al. [18] suggested two plots to evaluate the first condition of importance sampling. The first is a plot of the normalised weights (*w*_*j*_(*δ*)) against the estimates obtained from each imputed dataset under MAR $\left({\widehat{\theta}}_j\right)$, which enables the researcher to identify which of the imputed datasets takes a relatively high weight. The horizontal line in this plot is fixed at $\frac{1}{m}$, which represents the scenario where the imputations have the same weights (i.e. MAR mechanism (*δ* = 0)), and the vertical dashed line corresponds to the pooled MI estimate under MAR.

The second is a plot of the running weighted estimate under MNAR $\left({\widehat{\theta}}^{MNAR}\right)$ against the number of imputations (*m*), with the estimates obtained from each imputed dataset under MAR $\left({\widehat{\theta}}_j\right)$ presented on the right *y*-axis. If the running weighted MNAR estimate is heading towards the edge of the range of MI estimates or it contains some evident vertical steps, further attention is required to determine why this is the case.

Unfortunately, assessing the second condition of importance sampling is possible only in artificial or simulated examples, where the values of the missing observations are known. Some possible causes of failure of this condition will be addressed in the section “Explanation of the method failure”.

## Methods

In this section, the procedures for generating and analysing dataset(s) used in the paper are explained in detail.

In brief, we initially illustrate the weighting approach and the graphical diagnostics described above using a single simulated dataset, where the missing data in the outcome are weakly MNAR (i.e. small departure from MAR (*δ* =0.2)). Next, we comprehensively evaluate the performance of the weighting approach through simulation studies, where we examine whether by increasing the number of imputed datasets it is possible to obtain unbiased estimates for large (*δ* =1) to moderate (*δ =* 0.5) magnitudes of departure from the MAR assumption (as opposed to the weak departure from MAR presented in the previous example).

### Model for simulated data

We use the following models for generating data throughout the paper. In the first model, pairs of observations *X* and *Y* are generated from a bivariate normal distribution, with each variable having mean 0 and variance 1, and the correlation between the two equal to 0.5. *X* is a fully observed covariate but the values of the outcome *Y* are made missing under MNAR using the particular form of the logistic regression in Equation (3), i.e. *logit*[*Pr*(*R* = 1|*X*, *Y*)] = *α* + *γX* + *δY*. We assume that the target analysis of interest is to estimate the marginal mean of *Y* (*μ*) and the association between *Y* and *X*, represented by the coefficient for *X* (*β*_*1*_) in the linear regression model:

10 $$ Y={\beta}_0+{\beta}_1X+\varepsilon $$

In the complete data, the former parameter of interest is obtained by calculating the sample mean of *Y*, and the latter is estimated using the ordinary least squares method.

In the second model, we let *Y* be a binary outcome variable and *X* be a normally distributed covariate. Similar to the previous model, we set *Y* observations to missing under MNAR mechanism using Equation (3). For our target analysis, we estimate the marginal proportion of the outcome, as well as the regression coefficient (*Φ*_1_) in the logistic regression model:

11 $$ logit(Y)={\varPhi}_0+{\varPhi}_1X $$

In the fully observed dataset, we obtain the former parameter by calculating the sample proportion of *Y* (i.e. the proportion of successes in the sample (*Y* = 1)), and estimate the latter using the maximum-likelihood method.

### Procedures for generating a single dataset

In order to illustrate the weighting approach, we first generate a single dataset with 500 observations under the first model described above. We set *γ* = 1, *δ* = 0.2 and *α* = 0.12 in Equation (3), so that the probability of observing *Y* observations was equal to 0.5 and that the departure from MAR was minimal.

### Simulation procedures for generating 1000 datasets

We investigate the performance of the weighting approach by conducting a series of simulation studies. Using the first model for generating data described above, we simulated 1000 datasets of 100 observations. In the first scenario that we examine, we set *α* = 0, *γ* = 1 and *δ* = 1 (a relatively large departure from MAR), and in the second scenario, *α* = 0, *γ* = 0.8 and *δ* = 0.5 (a moderate departure from MAR) to achieve approximately 50 % missingness in the outcome *Y* for each of the 1000 simulated datasets.

In order to quantify how strongly the probability of missingness depends on *X* and *Y*, a Receiver Operating Characteristic (ROC) analysis was carried out [40]. The area under the ROC curve (AUROC) measures how strongly *X* and *Y* relate to *R*: AUROC = 0.5 indicates MCAR, while AUROC = 1 means that *X* and *Y* completely determine missingness. This analysis resulted in a mean AUROC of 0.84 for *δ* = 1, and 0.77 for *δ* = 0.5 over the 1000 simulations.

We conducted a similar simulation study using the second model for generating data described above, imposing a 50 % missing data rate on *Y*, where we set *α* = −0.4, *γ* = 1 and *δ* = 1 for the first scenario presenting a large departure from MAR, and *α* = −0.1, *γ* = 1 and *δ* = 0.5 for the second scenario presenting a moderate departure from MAR. The mean area under the curve estimated from the ROC analysis of the 1000 simulated datasets was 0.78 for *δ* =1, and 0.76 for *δ* = 0.5.

### Statistical approaches for handling missing data

The parameters of interest in our single simulated dataset and simulation studies were estimated using (i) complete case analysis, (ii) MI to account for the missing data under MAR, and (iii) the weighting approach to account for the missing data under MNAR.

#### i. Complete case analysis

In the complete case analysis individuals whose *Y* values were assigned to missing were excluded and the standard analysis was performed on records with observed *Y*’s (i.e. ~ 50 % of the total cases).

#### ii. Multiple imputation

Multiple imputation was implemented with *m* imputed datasets under the MAR assumption. Missing data in the outcome variable *Y* were imputed multiple times using a linear or logistic regression model on *X* with *m* = 5, 10, 50, 100, 500 and 1000 imputations. This wide range of *m*’s was selected to include low values of *m*, which were suggested in the early literature [16], up to much larger values over and above the current recommendation [41–43]. Importantly, as suggested by Carpenter and Kenward [6], the weighting approach requires *m* ≥ 50. Of note, for the single simulated dataset, we chose a moderate number of imputations *m* = 300 to impute the missing *Y* observations.

The imputation and analysis models were the same throughout this paper. Imputation was performed using ‘*mi impute regress*’ for a normally distributed outcome and ‘*mi impute logit’* for a binary outcome, and Rubin’s rules were implemented using ‘*mi estimate*’ in Stata version 12.1 [11].

#### iii. The weighting approach

Sensitivity analysis was performed using the weighting approach within the MI framework by re-weighting the estimates obtained from MI under MAR. The departure from the MAR mechanism used in the MNAR analysis was *δ* = 0.2 for the single simulated dataset and *δ* =1 and 0.5 for the simulation studies, as used for generating the data. Of note, we return later in the section “Graphical method for selecting *δ*” to discuss whether we are able to select plausible values for *δ* using a graphical method proposed by Héraud-Bousquet et al. [30].

The estimates of the parameters of interest for the full dataset (i.e. before assigning missing data to *Y*) were averaged over the 1000 simulations (i.e. ‘Full dataset (before deletion)’ in Tables 2, 3, 4 and 5). The performance of the different statistical approaches for handling missing data was examined by 1) comparing the average parameter estimate over the 1000 datasets with incomplete data to the true value used to generate the data (i.e. zero for the marginal mean and 0.5 for the measure of association) and 2) computing empirical Monte Carlo standard errors (i.e. the Monte Carlo standard deviation of the point estimate).

See Additional file 1: Figure S1 and Additional file 2: Figure S2 for a summary of the steps taken for conducting the simulation study for the normally distributed and the binary outcome, respectively.

## Results

### Illustration using a single simulated dataset

Complete case analysis and MI under MAR (with *m* = 300) were used for handling missing data and the weighting approach was performed as a sensitivity analysis under MNAR following MI (setting *δ* = 0.2). Table 1 shows the empirical mean and standard deviation of the estimated parameters of interest for the different methods of handling the missing data as well as for the full dataset before values of *Y* were set to missing. According to the table, the estimated marginal mean of *Y* (*μ*) and the coefficient *β*_*1*_ from the linear regression of *Y* on *X* in the full dataset are very close to their true values (0 and 0.5, respectively). Under the complete case analysis, these estimates are far away from the full data values with large differences mainly in the estimate of the marginal mean as expected. The absolute difference of the MI estimates under MAR from the estimated values of the parameters of interest from the full dataset are 0.129 (i.e. 2.93 standard errors) and 0.074 (i.e. 1.89 standard errors), for the marginal mean of *Y* and the coefficient *β*_*1*_, respectively. The estimate from the sensitivity analysis under MNAR is 0.022 for the marginal mean and 0.518 for the measure of association, which are quite close to the values from the full dataset. This occurs as a result of using the true value of *δ* in the analysis; however, this cannot be expected in practice because the value of *δ* is unknown*.*

**Table 1:** Estimates of the marginal mean of the normally distributed outcome variable and the regression coefficient under four analysis methods for a single simulated dataset (*n* = 500*, m =* 300*, δ* = 0.2)

	*μ*		*β*	
	Parameter estimate	SE	Parameter estimate	SE
Full dataset (before deletion)	−0.007	0.044	0.501	0.039
Complete Case Analysis	0.282	0.057	0.426	0.058
Multiple Imputation under MAR	0.122	0.058	0.427	0.058
Sensitivity analysis under MNAR	0.022	0.045	0.518	0.046

**Fig. 1:**
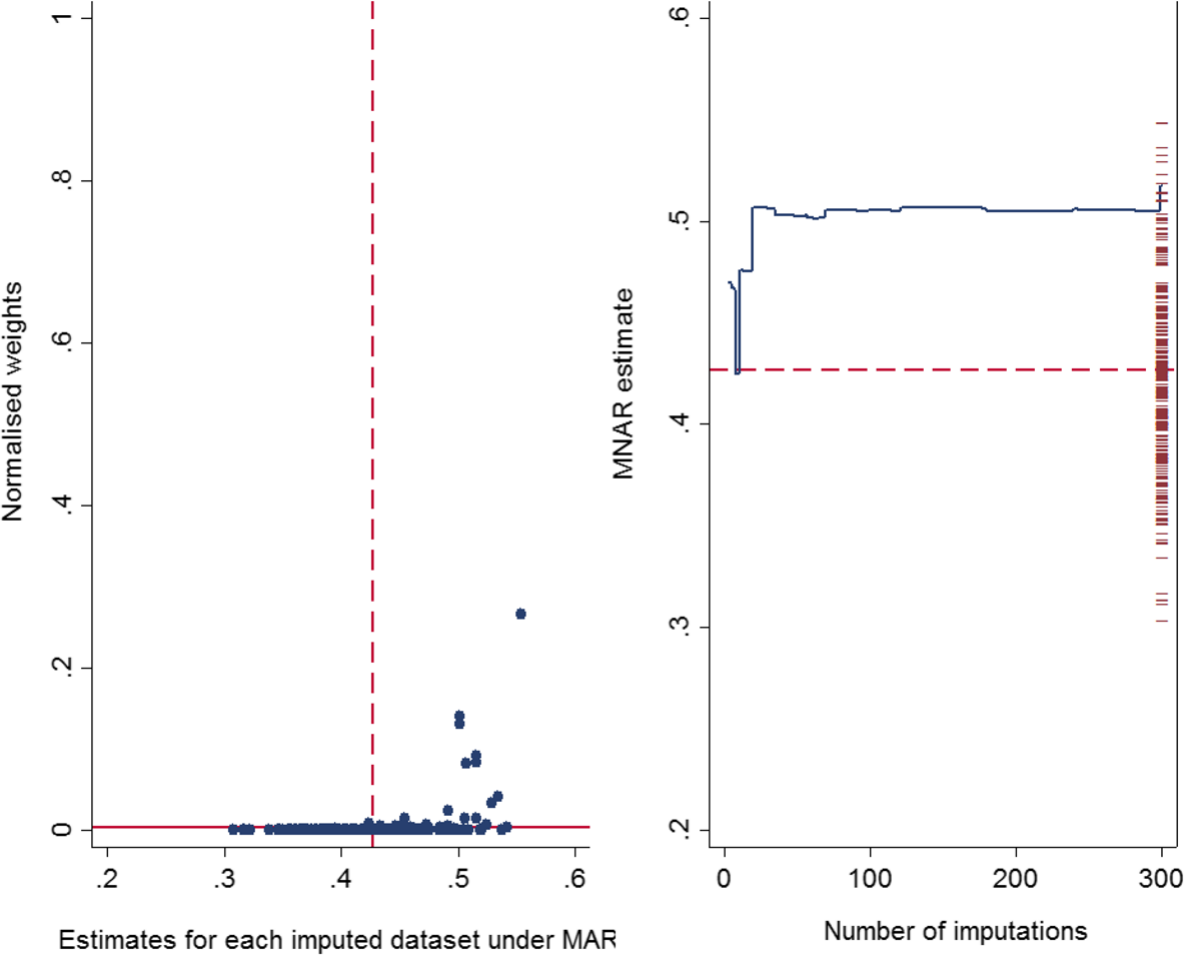
Graphical diagnostics (*n* = 500, *m* = 300, *δ*_(*True*)_ = 0.2, *β*_(*True*)_ = 0.5, ${\widehat{\beta}}_{(Simulate)}$ =0.501). Left panel: the normalised weight (calculated using Equation (7)) for each of the 300 imputed datasets, plotted against the estimated regression coefficient of *Y* on *X* obtained under MAR. The vertical dashed line corresponds to the pooled MI estimate under MAR. Right panel: the running mean of the MNAR estimate of the regression coefficient of *Y* on *X,* plotted against the number of imputed datasets. The estimates of the same coefficient obtained under MAR are plotted on the right *y*-axis for each of the 300 imputed datasets. The horizontal dashed line represents the pooled MI estimate under MAR

Graphical diagnostics explained earlier were applied in this single simulated dataset. As seen in Fig. 1, there is no extreme weight observed across the imputed datasets and the largest normalised weight is around 0.26 (left panel). In addition, the vertical drop observed in the running mean of the MNAR estimate at the early imputations appears to settle down as the number of imputations increases and the estimate is not at the edge of the range of the 300 MAR estimates (right panel). See Additional file 3: Figure S3, for graphical diagnostics for the marginal mean of the *Y* variable; for this parameter the running mean of the MNAR estimate is at the edge of the MAR estimates.

### Results of simulation experiment

The estimates of the marginal mean of the partially observed normally distributed outcome variable using complete case analysis, MI under MAR, and the sensitivity analysis using the weighting approach under MNAR are shown in Table 2. The table presents the averaged estimates across the 1000 simulated datasets for the different values of *m*. Note that since the estimates based on the full dataset and complete case analysis do not depend on the number of imputations, their corresponding results are shown only in the first column of the Table (i.e. *m* = 5).

**Table 2:** Estimates of the marginal mean of the normally distributed outcome variable under four analysis methods (*n* = 100, *δ* = 1); True value = 0

	Number of imputations (*m*)					
	5	10	50	100	500	1000
Full dataset (before deletion)	−0.003					
Complete Case Analysis	0.489					
Multiple Imputation under MAR	0.323	0.322	0.322	0.322	0.322	0.322
Sensitivity Analysis under MNAR	0.196	0.155	0.078	0.046	−0.018	−0.044

*Note:* The empirical Monte Carlo standard errors were all around 0.003 for MI and 0.004 for sensitivity analysis

According to Table 2, estimates obtained from the complete case analysis and MI under MAR overestimate the true value, as expected. The MNAR estimates of the marginal mean using the weighting method reduce as the number of imputations increase, but importantly do not converge to the value of the true mean as *m* increases.

Table 3 presents estimates of the regression coefficient obtained using the four methods. As seen in the table, the mean estimate of *β*_*1*_ across the 1000 simulated datasets for the full dataset is close to 0.5, as expected. The estimates for the complete case analysis and MI are similar and both are downwardly biased. The estimates under the MNAR sensitivity analysis increase with the number of imputations, and again do not converge to the true value of *β*_*1*_ as *m* → ∞. The results show that the absolute bias in the sensitivity analysis reduces to 0.028 (or ~5 %) after 10 imputations, but then rises with the number of imputations to a value of 0.135 (or ~27 %) for 1000 imputations.

**Table 3:** Estimates of the linear regression coefficient (*β*_*1*_) under four analysis methods (n = 100, *δ* = 1); True value = 0.5

	Number of imputations (*m*)					
	5	10	50	100	500	1000
Full dataset (before deletion)	0.498					
Complete Case Analysis	0.339					
Multiple Imputation under MAR	0.339	0.340	0.339	0.339	0.339	0.339
Sensitivity Analysis under MNAR	0.440	0.470	0.532	0.557	0.611	0.633

*Note*: The empirical Monte Carlo standard errors were all around 0.004 for MI and 0.005 for sensitivity analysis

**Fig. 2:**
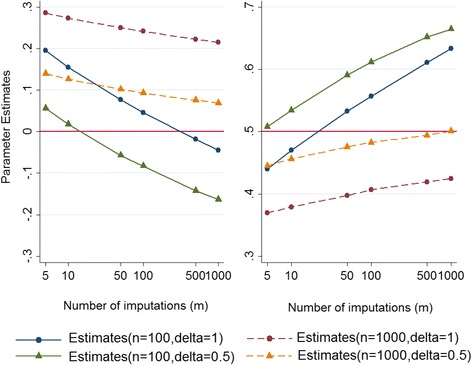
Estimates of the marginal mean (left panel) and the regression coefficient (right panel) for a normally distributed outcome obtained from the sensitivity analysis under MNAR against number of imputations (*m*) on a log scale

Further examination was carried out for estimating the marginal mean of the partially observed normally distributed outcome variable and the regression coefficient by 1) increasing the sample size of the simulated datasets from 100 to 1000; and 2) reducing the magnitude of departure from the MAR assumption (*δ*) from 1 to 0.5. A summary of the results for different scenarios is presented in the following graphs. According to the results presented in Fig. 2:

i. The parameter estimates decrease for the marginal mean and increase for the regression coefficient $\left({\widehat{\beta}}_1\right)$ as the number of imputations increases. The estimates appear to be converging to a single biased estimate (on the original scale); except potentially for the scenario with a large sample size (*n* = 1000) and a moderate departure from MAR (*δ* = 0.5). However, based on the observed patterns, it seems that by applying more imputations, the estimates obtained from a large *n* and moderate *δ* will move further away from the true value if *m* exceeds 1000.
ii. Surprisingly, the parameter estimates are lower for the marginal mean (left panel) and higher for the measure of association (right panel) for *δ* = 0.5 compared with *δ* = 1. It seems that we are observing two opposing effects here. One possible explanation for this observation might be the fact that while increasing *δ* from 0.5 to 1 in the MNAR analysis increases the potential for extreme weights, increasing *δ* in the data generating mechanism reduces the left-hand tail of the observed outcome distribution and thus, reduces the potential for extreme weights.

### Further investigation

We extended our simulation study to account for a partially observed binary outcome variable and a fully observed continuous covariate. The estimates of the marginal proportion of the binary outcome and the regression coefficient obtained from complete case analysis, MI under MAR, and the sensitivity analysis using the weighting approach under MNAR are summarised in Tables 4 and 5, respectively. It is apparent from Tables 4 and 5 that the results of the complete case analysis and MI under MAR are biased, as expected. Again the MNAR estimates decrease for estimating the marginal proportion of the outcome variable as the number of imputations increase and do not converge towards the true value of the parameter (Table 4). Also, the estimates from the sensitivity analysis for the regression coefficient increase as the number of imputations increases, thus moving further away from the true value (Table 5).

**Table 4:** Estimates of the marginal proportion of the binary outcome variable under four analysis methods (*n* = 100*, δ* = 1); True value = 0*.*5

	Number of imputations (*m*)					
	5	10	50	100	500	1000
Full dataset (before deletion)	0.497					
Complete Case Analysis	0.639					
Multiple Imputation under MAR	0.608	0.608	0.608	0.608	0.608	0.608
Sensitivity Analysis under MNAR	0.545	0.526	0.493	0.482	0.460	0.452

*Note*: The empirical Monte Carlo standard errors were all around 0.002 for MI and for sensitivity analysis

**Table 5:** Estimates of the logistic regression coefficient (*φ*_*1*_) under four analysis methods (*n* = 100*, δ* = 1); True value = 0.5

	Number of imputations (*m*)					
	5	10	50	100	500	1000
Full dataset (before deletion)	0.524					
Complete Case Analysis	0.329					
Multiple Imputation under MAR	0.331	0.332	0.331	0.331	0.332	0.332
Sensitivity Analysis under MNAR	0.546	0.601	0.693	0.727	0.781	0.797

*Note*: The empirical Monte Carlo standard errors were all around 0.008 for MI for sensitivity analysis

**Fig. 3:**
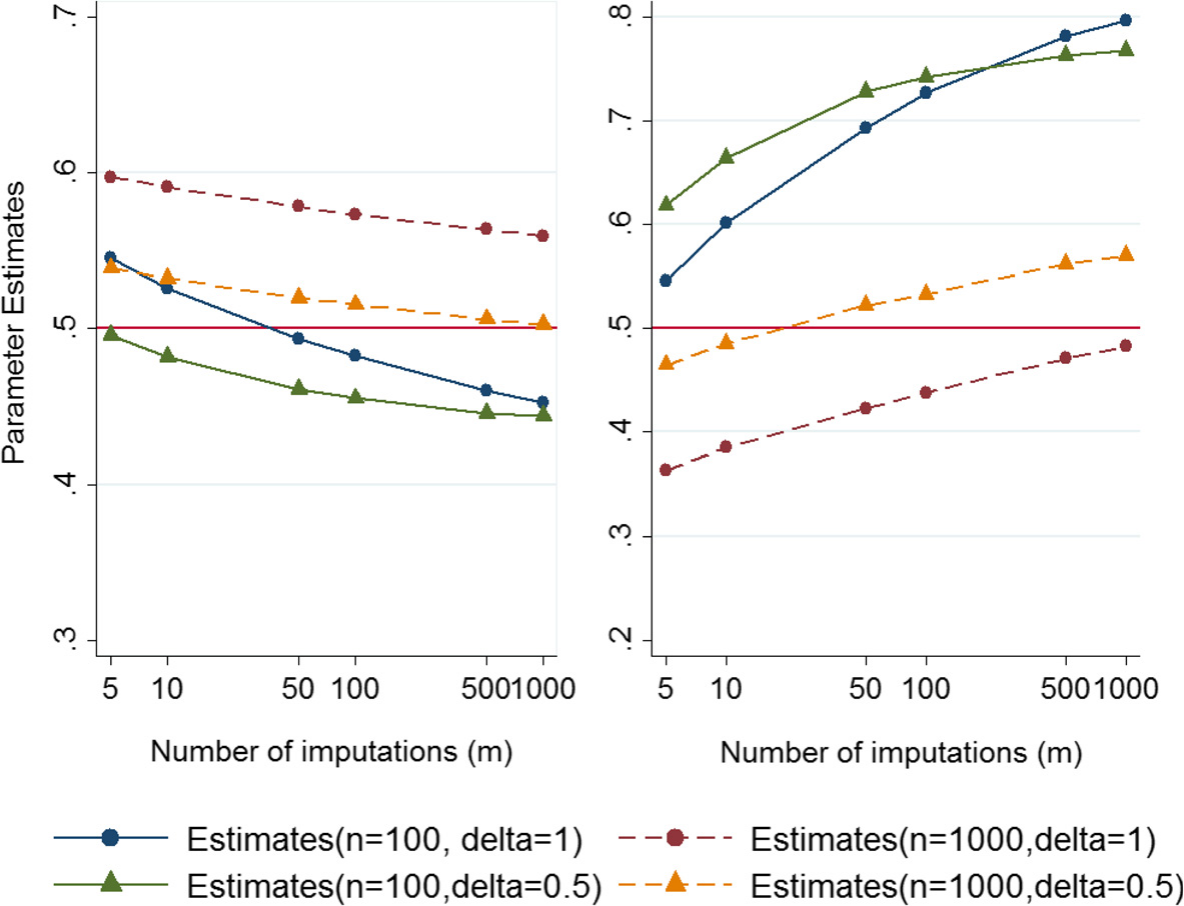
Estimates of the marginal proportion and the logistic regression coefficient obtained from the sensitivity analysis under MNAR against number of imputations (*m*) for a binary outcome

Additional examination was carried out when the sample size of the simulated data was increased from 100 to 1000 and the degree of departure from MAR reduced from 1 to 0.5. Again the results show biased estimates using complete case analysis and MI under MAR for both the marginal proportion of the partially observed outcome and the measure of association. Figure 3 presents the parameter estimates of interest under MNAR (left panel: marginal proportion of the outcome variable and right panel: exposure-outcome relationship) against the number of imputations, when the sample sizes are 100 and 1000 and *δ* are 1 and 0.5. The Figure illustrates almost the same pattern as Fig. 2; that is, the MNAR estimates do not approach the true parameter values as *m* → ∞.

### Explanation of the method failure

In this section, we discuss further details of the weighting approach within the MI framework, which may explain why the weighting approach fails, as observed in the previous section.

Consider imputation in the setting described under the first model in the “Methods” section (i.e. normally distributed outcome (partially observed) and covariate (fully observed)). Initially, the linear regression model, *Y* = *β*_0_ + *β*_1_*X* + *ε*; (*ε* ∼ *N*(0, *σ*^2^)), is fitted to the observed data in order to obtain the point estimates of $\widehat{\beta}$ (i.e. ${\widehat{\beta}}_0$ And ${\widehat{\beta}}_1$ in this example) and ${\widehat{\sigma}}^2$. Then, a new parameter estimate β_*_ and its associated variance (σ_*_^2^) are drawn from their joint posterior distribution in two steps:

12 $$ {\sigma_{\ast}}^2\sim {\widehat{\sigma}}^2\frac{\left({n}_o-q\right)}{\chi_{n_o-q}^2} $$

13 $$ {\beta}_{*}\left|{\sigma_{*}}^2\right.\sim N\left(\widehat{\beta},{\sigma_{*}}^2{\left({X}_o^{\prime }{X}_o\right)}^{-1}\right) $$

where *n*_0_ denotes the number of complete cases (i.e. observed data values for *Y* in this example), *q* is the number of parameters in the linear regression model (i.e. two for this simulation example) and *X*_*o*_ corresponds to the *X* values where the *Y* values are also observed (i.e. the complete cases) [6].

One reason why the weighting approach may fail is because the posterior predictive distribution of *Y* given *X*, from which the imputations for this example are drawn, is a Student-*t* distribution. This is because the imputation parameter σ_*_^2^ follows a scaled inverse chi-squared distribution, shown in Equation (12), and the true variance is unknown. Importantly, the tails of the probability density function of the *t*-distribution follow a power function. Against that, the weights have the form of an exponential function (refer to Equation (6)); thus, in the tail of the distribution of the imputed values ${\varSigma}_{i\in {I}_Y}{Y}_{ij}$, the weights increase more quickly than the density of the distribution decreases. It can be shown that, across the imputed datasets and given the observed data, the sum of *Y*_*i*_ in Equation (6) (i.e. ${\varSigma}_{i\in {I}_Y}{Y}_{ij}$, where *i* indicates the *i*^*th*^ imputed value and *j* represents *j*^*th*^ imputed dataset) itself has a *t*-distribution with *n*_0_-*q* degrees of freedom, where *n*_0_ is the number of observed values. This links more closely the shape of the imputation distribution to the shape of the weight function. Consequently, the second condition of the importance sampling is violated as the weight, the ratio of the imputation distribution under MNAR to the imputation distribution under MAR (g/*f* described in the “Importance sampling” section), is unbounded. This results in an inconsistent MNAR estimate, and may explain why the weighting approach fails in our example.

Note that the explanation above does not apply to a binary outcome variable, since the imputation distribution is no longer a *t*-distribution. In fact, β_*_ is drawn from the asymptotic approximation to its posterior distribution, and the imputed values are only approximate draws from the posterior predictive distribution of the missing data. However, in many cases, this asymptotic approximation may not be an accurate approximation to the joint posterior distribution, as it might be extremely wrong out in the tails of the distribution. Thus, again in the context of an incomplete binary variable it appears that the importance weights become unbounded and the MNAR estimate may remain unstable.

It is worth mentioning that the *t*-distribution is similar to the normal distribution, but with heavier tails for small sample sizes. As the sample size increases, the degrees of freedom (*df*) increases and the *t*-distribution approaches the normal distribution. For datasets that are small or the number of missing values is large, the missing observations are drawn from a *t*-distribution with heavy tails. As a result, the MNAR estimate becomes even more unstable in these scenarios since, across imputed datasets, the MAR estimate may become noisier and improperly weighted. Of note, the problem of unbounded weights is not restricted to small sample sizes; but it becomes more evident when the sample size is small since the imputed values are drawn from much heavier tails of the posterior predictive distribution of missing data. Hence, this issue extends to all datasets irrespective of sample size.

In general, it seems that as we increase the number of imputations, the more likely we are to draw a really extreme imputed dataset which is assigned nearly all the weight. The problem arises because the weight used for calculating the overall MNAR estimate is not actually the ratio of *t*-densities, and thus, the ratio is definitely unbounded unless the *Y*_*i*_’s are bounded. In fact, this problem occurs as a result of a failure in the argument of Carpenter et al. [18]. In their paper it was shown that the importance ratio, which was described in the “Importance sampling” section, is

14 $$ \frac{g}{f}=\frac{f\left[Y\left|X,R=0\right.\right]}{f\left[Y\left|X,R=1\right.\right]}=\frac{f\left[R=0\left|Y,X\right.\right]}{f\left[R=1\left|Y,X\right.\right]}\times \frac{f\left[X,R=1\right]}{f\left[X,R=0\right]} $$

where, in a simple scenario, there was only one individual with a fully observed covariate *X*, a partially observed response *Y*, and a missingness indicator *R*, which was zero if *Y* was missing. It was claimed that under the logistic model in Equation (3), *f*[*R* = 1|*Y*, *X*] equates to expit(*α* + *γX* + *δY*), and thus the importance ratio was simplified as $\exp \left\{-\left[\alpha +\gamma X+\delta Y\right]\right\}\frac{f\left[X,R=1\right]}{f\left[X,R=0\right]}\propto exp\left(-\delta Y\right)$. However, this simplification relies on the assumption that all the parameters are known. A correct weighting would compute *f*[*R* = *r*|*Y*, *X*], where r = 0,1 by integrating *f*[*R* = *r*|*Y*, *X*, *α*, *γ*, *δ*] over the posterior distribution of *α* and *γ* in the numerator and denominator in Equation (14).

In the more general case of imputation models that are GLMs (e.g. logistic regression), where the imputation models make a normal approximation to the posterior, it seems that the weighting method will also fail because of the reason mentioned above. However, this could potentially be avoided for binary variables with missing data by applying bootstrapping in the imputation process. The idea is to draw a single bootstrap sample (i.e. random sampling with replacement) from the data (multiple times) and fitting the imputation model to the bootstrap sample in order to avoid situations where the asymptotic approximation may be inadequate for the posterior distribution [44].

It seems that the method failure is likely to occur more obviously in smaller samples, since they have smaller degrees of freedom. Also, the bias in the MNAR estimate will probably increase as the number of imputations increases in smaller datasets. Furthermore, the largest weight will increase as the number of imputations increases and the MNAR estimate will become unstable (because the chance of observing an imputed dataset with the minimal sum of the imputed values (for *δ* > 0), or with a maximal sum of imputed values (for *δ* < 0) increases as *m* increases).

### Graphical method for selecting *δ*

In the simulation studies described earlier, we considered an unrealistic situation where we assumed that the value of *δ* was known. In this section, we describe a real situation where the value of *δ* is unknown, and then apply the graphical method proposed by Héraud-Bousquet et al. [30] to select a range of plausible values for *δ.*

### Overview of procedure for choosing *δ*

In the absence of sufficient information about the unmeasured factors in a dataset, it is not typically possible to estimate the degree of departures from the MAR mechanism for performing a sensitivity analysis in practice.

One way to select the magnitude of departures from MAR is to elicit all possible values that would be considered reasonable by experts. Héraud-Bousquet et al. [30] have recently developed a graphical method for obtaining a range of plausible values of *δ* which represent local departures from the MAR assumption. This graphical method was illustrated in four steps using epidemiological data from an observational cohort, in which normalised weights for each imputed dataset were plotted against different possible values of *δ*.

According to Héraud-Bousquet et al.’s suggestion for obtaining a range of *δ*, the maximum normalised weight should be around 0.5, and at least five normalised weights should be above $\frac{1}{m}$. These rules are then used to select a range of values for *δ*. Under this approach the sign of *δ* is identified according to the experts’ opinions and previous experiences. In the next section, this graphical method will be applied to the single simulated dataset described earlier, where data are MNAR (*δ* = 0.2), and then will be used in two further real examples with larger degrees of MNAR (*δ* = 0.5 and 1). The aim is to determine a proper range of plausible *δ* values and to examine whether the selected range captures the true value of *δ*.

### Illustration using a single simulated dataset *(continued)*

**Fig. 4:**
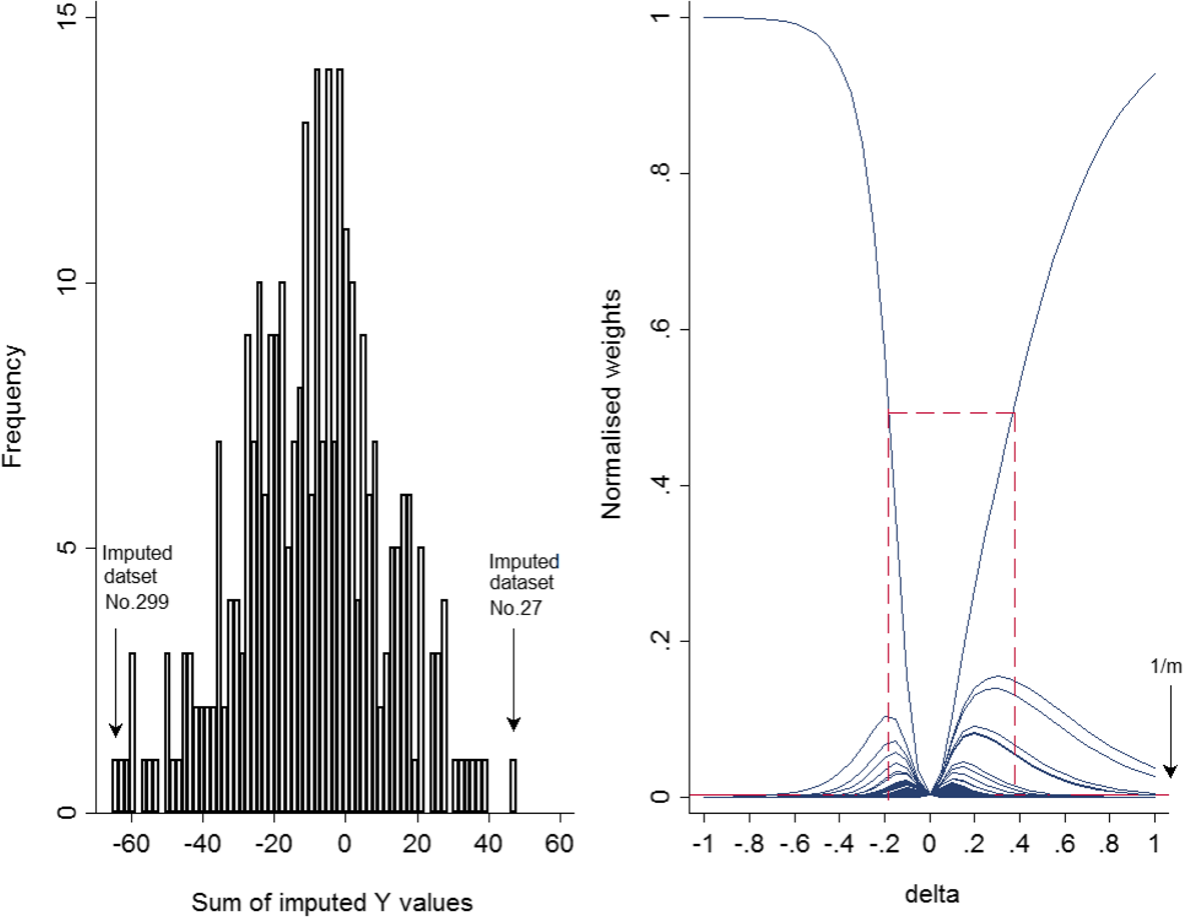
Graphical procedure for selecting a range for *δ*, *n* = 500, *m* = 300, δ_(*True*)_ = 0.2. Left panel: histogram of the sum of imputed *Y* values for each data set. Right panel: graphical determination of delta for the variable *Y* using the approach suggested by Héraud-Bousquet et al. [30]

We first start with the single simulated data example described earlier and apply the graphical method for choosing a range for *δ*, and then we extend our example to larger magnitudes of *δ*. Figure 4 shows a histogram of the sum of imputed *Y* values in each of 300 imputed datasets (left panel) and the normalised weights based on different *δ* values (right panel), where each curve is plotted as a function of *δ*, with a different function for each of the 300 imputed datasets.

Héraud-Bousquet et al. [30] mentioned that the maximum normalised weight across the imputed datasets corresponds to the imputed dataset with the minimum sum of the imputed values when *δ* is positive (*δ* > 0), or the maximum sum of the imputed values when *δ* is negative (*δ* < 0). As can be seen, the datasets no. 299 and 27 have the minimum and maximum sum of imputations, respectively (refer to the left panel of Fig. 4). Since the true value of *δ* is 0.2 in this dataset, the maximum normalised weight corresponds to the dataset no. 299 since this has the minimum sum of imputed values.

The right panel of Fig. 4 presents the normalised weights against different *δ* values. According to Héraud-Bousquet, et al., the maximum normalised weight should be around 0.5, and more than 5 normalised weights should be above the line of $\frac{1}{300}$. The range of *δ* values that fit these criteria is shown by dashed lines. This range includes values of *δ* between −0.18 and 0.38 and captures the true value of *δ* (0.2) used to simulate the data.

**Fig. 5:**
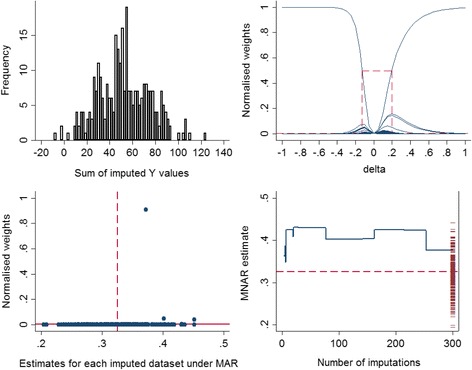
Graphical procedure for selecting a range for *δ* (top). Top left panel: histogram of the sum of imputed *Y* values for each dataset. Top right panel: graphical determination of delta for the variable *Y* using the approach suggested by Héraud-Bousquet et al. [30]. Graphical diagnostics(bottom). Bottom left panel: normalised weights against the MAR estimates obtained from each of the 300 imputed dataset. Bottom right panel: mean of the MNAR estimate against the number of imputations (*n* = 500, *m* = 300, δ_(*True*)_ = 1, *β*_(*True*)_ = 0.5, ${\widehat{\beta}}_{(Simulate)}$ =0.501)

Further investigation was carried out by increasing the magnitude of *δ* in the simulated data to 0.5 and 1, with everything else identical. Figure 5 shows the graphical procedure for selecting a range for *δ* and graphical diagnostics explained in the section “Graphical diagnostics”, for the measure of association when *n* = 500, *m* = 300 and the true *δ* is 1.

It is apparent that the range for *δ* obtained from the plot above (−0.13, 0.2) does not capture the true value of *δ* =1 for this dataset (top right panel). There is a single imputed dataset which has a high weight, meaning that this imputed dataset is very influential. Further examination showed that about 99 % of the weight was concentrated on this single imputed dataset, which corresponds to the outlying weight in the bottom left panel of Fig. 5, and the large vertical drop at 253 imputed datasets in the bottom right panel of Fig. 5. See Additional file 4: Table S1 for estimates of the parameters of interest, with 95 % CI and SE, and Additional file 5: Figure S4, for the graphical diagnostics for the marginal mean of the *Y* variable.

The same results were observed when the moderate magnitude of departure from MAR was selected (*δ* = 0.5) (see Additional file 6: Table S2, Additional file 7: Figure S5 and Additional file 8: Figure S6). Surprisingly, even when the sample size was increased from 500 to 1000, with a small value of departure *δ* = 0.2 one imputed dataset was again given a very large weight (see Additional file 9: Table S3, Additional file 10: Figure S7 and Additional file 11: Figure S8).

## Discussion

MI is a common approach for handling missing data. Standard implementation of MI assumes the data are MAR, so it is widely recommended to perform a sensitivity analysis to explore the robustness of inferences to departures from the MAR assumption.

This study evaluated a selection-model-based weighting approach for performing a sensitivity analysis within the MI framework. Simulation studies were designed to assess whether the proposed method can provide unbiased MNAR estimates across varying numbers of imputations and sample sizes where the magnitude of departures from MAR varied from moderate to large. The results indicate that, in general, the weighting approach produces highly unstable MNAR estimates across varying numbers of imputations. This study also evaluated the graphical method proposed by Héraud-Bousquet et al. [30] for obtaining a range of plausible values of the sensitivity parameter *δ* (i.e. the magnitude of departure from the MAR assumption) to examine whether the plausible value is far away from the true value.

Carpenter et al. [18] introduced the weighting approach and showed that, in a single simulation study, this method can gradually remove the bias in the MNAR estimate as the number of imputations increases. Further, Carpenter and Kenward [6] noted that if the dataset is small or contains a large number of missing values, by increasing the number of imputations the MNAR estimate obtained from the weighting approach might be unstable; however, if a suitable imputation model is chosen the method will perform well in small datasets. A partial solution has been recently developed when performing the weighting approach in small datasets (personal communication: James Carpenter and Melanie Smuk).

In a different application of the weighting approach where MI followed by re-weighting was used to assess the sensitivity of the pooled estimate in a meta-analysis to selection bias, Carpenter et al. [45] commented on how the importance ratio will become unbounded as selection bias of studies included in the meta-analysis increases. In their paper they proposed a correction to the weights formula and illustrated that this performed well when there was a moderate selection bias and more than 10 observed studies included in the meta-analysis.

The simulation study that was carried out by Carpenter et al. [18] was the same as our study described in the “Methods” section (*n* = 100, *δ* = 1) where the marginal mean of a partially observed continuous outcome variable was the parameter of interest. We extended their simulation study to investigate the additional parameter, the regression coefficient for the outcome on the exposure variable. In our simulation studies, we identified that the MNAR estimates were biased and did not converge to the true value of the parameters of interest for either the marginal mean of the outcome or the regression coefficient as the number of imputations increased. A similar pattern was also apparent in the simulation study by Carpenter et al. [18], where at 1000 imputations the MNAR estimate was negative (−0.01), and based on our findings, we expect this estimate would move further away from zero if more than 1000 imputations were performed. Of note, there is a small discrepancy observed between our MNAR estimates and the original article by Carpenter et al. [18], over increasing number of imputations (≥50 imputations). One explanation for these discrepancies is stochastic variability in the imputation process that increases with the number of imputations. That is, the random draws of the imputation model parameters from their posterior distributions for creating the imputed values. In particular, increasing the number of imputations increases the chance of drawing an extreme imputed dataset that is assigned an extreme weight. Consequently, the MNAR estimate becomes highly dependent on that single imputed dataset as the number of imputations increases, and thus the distribution of the MNAR estimates over 1000 imputations has wider tails than the distribution over fewer imputations. Our investigation shows that the distribution of simulated point estimates is heavy-tailed in such a way that Normal-theory confidence intervals fail. Therefore, in such cases, Monte Carlo errors may be a poor guide to simulation error.

Biased estimates were also observed when we increased the sample size to 1000, explored a moderate departure in the MAR assumption (*δ* = 0.5), and extended our evaluation to a binary (partially observed) outcome variable.

The findings of our investigation highlight that the weights used in estimating the overall parameter estimate under MNAR become unbounded as the number of imputations increases. This leads to improper weighting of the imputed datasets so that one or two datasets take approximately all the weight. It was shown that the problem of large weights occurs not only for large departure from MAR, but it also may occur for small and moderate departures even in large datasets. As explained in the section “Explanation of the method failure”, the problem arises from the computation of the weights, and this method should work better if the weights were correctly computed. However, this issue may not have a simple solution that is computationally convenient.

The graphical method proposed by Héraud-Bousquet, et al. [30] for selecting *δ* relies heavily on the (normalised) weights, which themselves depend on the imputed values under MAR. This limits the usefulness of this approach. By definition, we cannot estimate *δ* from the data at hand; hence, obtaining a range for *δ* based on the available data seems inherently implausible. In fact, our findings demonstrate that this method does not perform adequately as a graphical approach for selecting a range of *δ* did not capture the true value of *δ* used in our simulation studies. Unfortunately, satisfactory guidelines are not currently available in the literature regarding the selection of *δ* for performing sensitivity analyses via MI, and further research is required to develop strategies if this is going to be a worthwhile avenue to pursue. The only principled approach to determine clinically plausible values of *δ* is to elicit these from expert knowledge informed as much as possible from external empirical evidence [35, 37]. This approach has been adopted in other areas of statistics [46, 47].

An alternative approach to assess the impact of departure from the MAR assumption within the MI framework is the pattern-mixture approach [6, 20, 21, 35]. This method is straightforward and easier to comprehend for non-statistical collaborators compared with the weighting approach [29]. Under the pattern-mixture approach, the degree of departure from MAR is defined as the difference (shift) in the mean of a partially observed variable between the unobserved and observed data. Within the MI framework, this alternative approach is applicable for both partially observed outcomes and covariates, and potentially when more than one variable has missing data. In the case of a continuous partially observed variable, missing data are imputed using standard MI assuming MAR, and then the imputed values in each imputed datasets are shifted (i.e. add or multiply *δ*_*pm*_, a pattern-mixture model sensitivity parameter, to each of the imputed values) in such a way that they represent the MNAR mechanism. When there is a partially observed binary variable, the shift of *δ*_*pm*_ needs to be added to the imputation model; therefore the missing values are, in fact, drawn from an imputation model assuming MNAR rather than MAR. More technical details of this approach are provided by Carpenter and Kenward [6], Ratitch et al. [20], Siddique et al. [48, 49], and White et al. [35].

## Conclusions

In summary, in the examples studied, although the weighting approach outperformed the MAR approach it still suffered from bias. Importantly, the current study demonstrates that the weighting approach fails to obtain unbiased estimates for parameters of interest in a very simple bivariate model when data are MNAR, even when using as many as 1000 imputations. The present findings suggest that this method will produce biased parameter estimates as long as the weights are obtained using the formula proposed by Carpenter et al. [18]. This potential for bias using the weights proposed by Carpenter et al. should be recognised by users, and more appropriate methods should be developed. Hence, additional investigation into MNAR approaches perhaps with more focus on the pattern-mixture approach as an alternative method for conducting a sensitivity analysis following MI is desirable.

## Additional files

Additional file 1: Figure S1. Procedure for performing a simulation study for a normally distributed outcome. (DOCX 31 kb) Additional file 2: Figure S2. Procedure for performing a simulation study for a binary outcome variable. (DOCX 31 kb) Additional file 3: Figure S3. Graphical diagnostics for a single simulated dataset (*n* = 500, *m* = 300, *δ*_(*True*)_ = 0.2, *μ*_(*True*)_ = 0, ${\widehat{\mu}}_{(Simulate)}$ = − 0.007). (DOCX 2362 kb) Additional file 4: Table S1. Estimates of the marginal mean of the normally distributed outcome variable and the regression coefficient under four analysis methods for a single simulated dataset *(n* = 500*, m =* 300*, δ* = 1). (DOCX 18 kb) Additional file 5: Figure S4. Graphical diagnostics for a single simulated dataset (*n* = 500, *m* = 300, *δ*_(*True*)_ = 1, *μ*_*(True)*_ = 0, ${\widehat{\mu}}_{(Simulate)}$ = − 0.007). (DOCX 2362 kb) Additional file 6: Table S2. Estimates of the marginal mean of the normally distributed outcome variable and the regression coefficient under four analysis methods for a single simulated dataset *(n* = 500*, m =* 300*, δ* = 0.5). (DOCX 17 kb) Additional file 7: Figure S5. Graphical procedure for selecting a range for *δ* (top). Top left panel: histogram of the sum of imputed *Y* values for each data set. Top right panel: graphical determination of delta for the variable *Y* using the approach suggested by Héraud-Bousquet et al. Graphical diagnostics (bottom). Bottom left panel: normalised weights against the MAR estimates obtained from each of the 500 imputed dataset. Bottom right panel: mean of the MNAR estimate against the number of imputations (*n* = 500, *m* = 300, *δ*_(*True*)_ = 0.5, *β*_(*True*)_ = 0.5, ${\widehat{\beta}}_{(Simulate)}$ =0.501). (DOCX 2362 kb) Additional file 8: Figure S6. Graphical diagnostics for a single simulated dataset (*n* = 500, *m* = 300, *δ*_(*True*)_ =0.5, *μ*_(*True*)_ =0, ${\widehat{\mu}}_{(Simulate)}$ = − 0.007). (DOCX 2362 kb) Additional file 9: Table S3. Estimates of the marginal mean of the normally distributed outcome variable and the regression coefficient under four analysis methods for a single simulated dataset *(n* = 1000*, m =* 500*, δ* = 0.2). (DOCX 17 kb) Additional file 10: Figure S7. Graphical procedure for selecting a range for *δ* (top). Top left panel: histogram of the sum of imputed *Y* values for each data set. Top right panel: graphical determination of delta for the variable *Y* using the approach suggested by Héraud-Bousquet et al. Graphical diagnostics (bottom). Bottom left panel: normalised weights against the MAR estimates obtained from each of the 500 imputed dataset. Bottom right panel: mean of the MNAR estimate against the number of imputations (*n* = 1000, *m* = 500, *δ*_(*True*)_ = 0.2, *β*_(*True*)_ = 0.5, ${\widehat{\beta}}_{(Simulate)}$ =0.498). (DOCX 2362 kb) Additional file 11: Figure S8. Graphical diagnostics for a single simulated dataset (*n* = 1000, *m* = 500, *δ*_(*True*)_ = 0.2, *μ*_(*True*)_ = 0, ${\widehat{\boldsymbol{\mu}}}_{\left(\boldsymbol{Simulate}\right)}$ = − 0.009). (DOCX 2362 kb)

## Abbreviations

CC: Complete case
DF: Degrees of freedom
MAR: Missing at random
MCAR: Missing completely at random
MI: Multiple imputation
MNAR: Missing not at random
SE: Standard Error
