# Supplementary material for: Evaluation of a weighting approach for performing sensitivity analysis after multiple imputation
Source: BMC Med Res Methodol. 2015 Oct 13;15:83. doi: 10.1186/s12874-015-0074-2 (PMC4604630; doi:10.1186/s12874-015-0074-2)
Supplement: Additional file 1: Figure S1. — Procedure for performing a simulation study for a normally distributed outcome. (DOCX 31 kb) [file 12874_2015_74_MOESM1_ESM.docx]

| **Step 2: Assignment of missing data**   - Set approximately 50% of the observations of $Y$ to missing under MNAR based on $logit\left[ \Pr\left( R=1\vert X, Y \right) \right]=\alpha+\gamma X+\delta Y$, where the missingness indicator $R$=1 if $Y$ is observed and $R=$0 otherwise; for   *n*=100 – ($\alpha=0$, $\gamma=1$ and$\delta=1$) and ($\alpha=0$, $\gamma=0.8$ and$\delta=0.5$ )  *n*=1000 – ($\alpha=0$, $\gamma=1$ and$\delta=1$) and ($\alpha=0$, $\gamma=1$ and$\delta=0.5$ )  **Step 3: Target analysis**   1. *Estimated parameters of interest*    - Marginal mean of the normally distributed outcome    - Exposure-outcome relationship ($\beta_{1}$) using: $Y=\beta_{0}+\beta_{1} X+ɛ$. 2. *Statistical approaches for handling missing data*    - Complete case analysis    - MI under MAR (using ‘*mi impute regress’*)    - MI under MNAR (using the weighting approach) |
| --- |

**Step 1: Generating the 1000 datasets of size 100/1000**

- Covariate $X$ and outcome $Y$ generated from a bivariate normal distribution, with each having mean 0 and variance 1, and the correlation between the two equal to 0.5.
