# Supplementary material for: Evaluation of a weighting approach for performing sensitivity analysis after multiple imputation
Source: BMC Med Res Methodol. 2015 Oct 13;15:83. doi: 10.1186/s12874-015-0074-2 (PMC4604630; doi:10.1186/s12874-015-0074-2)
Supplement: Additional file 2: Figure S2. — Procedure for performing a simulation study for a binary outcome variable. (DOCX 31 kb) [file 12874_2015_74_MOESM2_ESM.docx]

| **Step 3: Target analysis**   1. *Estimated parameters of interest*    - Marginal proportion of the binary outcome    - Exposure-outcome relationship ($\phi_{1}$) using: $logit(Y)=\phi_{0}+\phi_{1} X$ 2. *Statistical approaches for handling missing data*    - Complete case analysis    - MI under MAR (using ‘*mi impute logit’*)    - MI under MNAR (using the weighting approach)   **Step 1: Generating the 1000 datasets of size 100/1000**   - Covariate: $X\sim N (0,1)$ - Outcome $Y$ dependent on $X$:   $logit\left[ \Pr\left( Y=1\vert X \right) \right]=\alpha_{0}+\alpha_{1} X$, where $\alpha_{0}=0$, $\alpha_{1}=0.5$. $\to Y\sim Binom (1,p)$  **Step 2: Assignment of missing data**   - Set approximately 50% of the observations of $Y$ to missing under MNAR based on $logit\left[ \Pr\left( R=1\vert X, Y \right) \right]=\alpha+\gamma X+\delta Y$, where the missingness indicator $R$=1 if $Y$ is observed and $R=$0 otherwise; for   *n*=100 – ($\alpha=-0.4$, $\gamma=1$ and$\delta=1$ ) and ($\alpha=-0.1$, $\gamma=1$ and$\delta=0.5$ )  *n*=1000 – ($\alpha=-0.5$, $\gamma=1$ and$\delta=1$ ) and ($\alpha=-0.26$, $\gamma=1$ and$\delta=0.5$ ) |
| --- |
