# Supplementary figures and images for: Evaluation of a weighting approach for performing sensitivity analysis after multiple imputation
Source: BMC Med Res Methodol. 2015 Oct 13;15:83. doi: 10.1186/s12874-015-0074-2 (PMC4604630; doi:10.1186/s12874-015-0074-2)

**
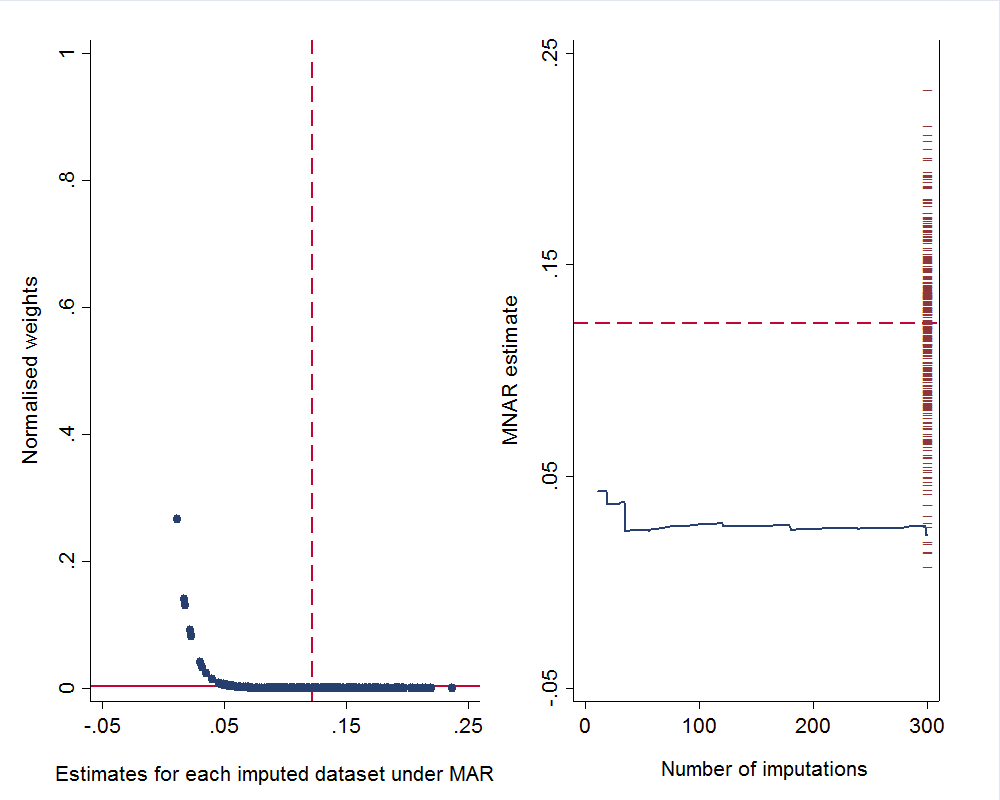
**

Supplement: Additional file 3: Figure S3. — Graphical diagnostics for a single simulated dataset (n = 500, m = 300, δ (True) = 0.2, μ (True) = 0, ${\widehat{\mu}}_{(Simulate)}$μ^(Simulate) = − 0.007). (DOCX 2362 kb) [file 12874_2015_74_MOESM3_ESM.docx]

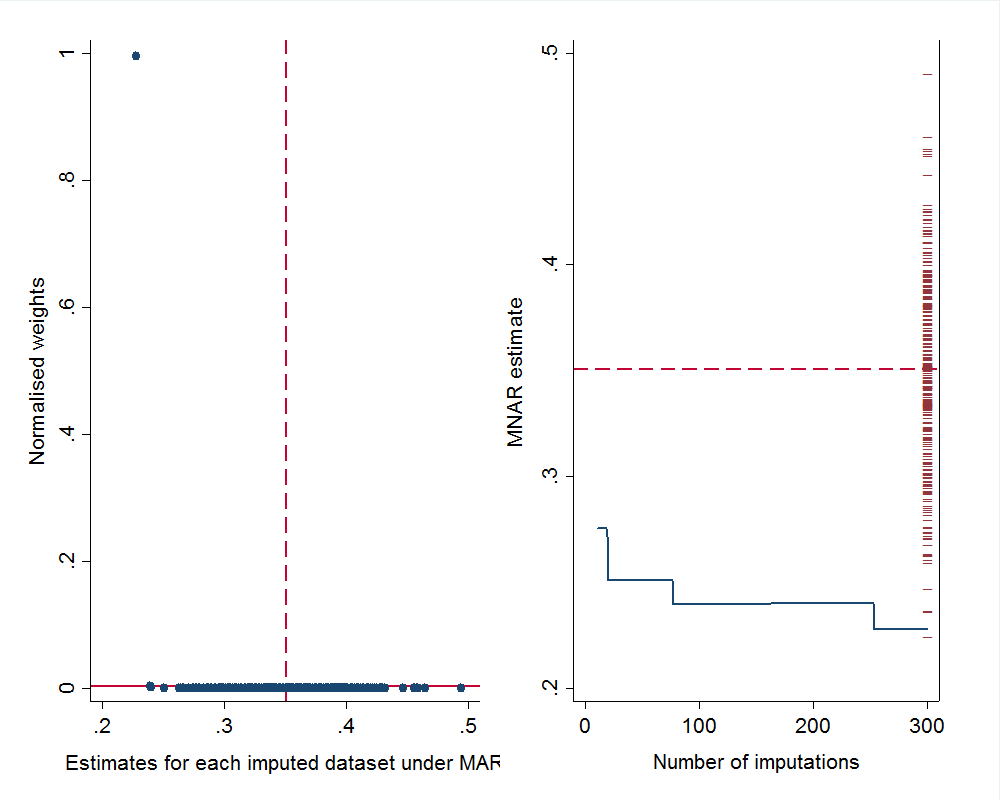

Supplement: Additional file 5: Figure S4. — Graphical diagnostics for a single simulated dataset (n = 500, m = 300, δ (True) = 1, μ (True) = 0, ${\widehat{\mu}}_{(Simulate)}$μ^Simulate = − 0.007). (DOCX 2362 kb) [file 12874_2015_74_MOESM5_ESM.docx]

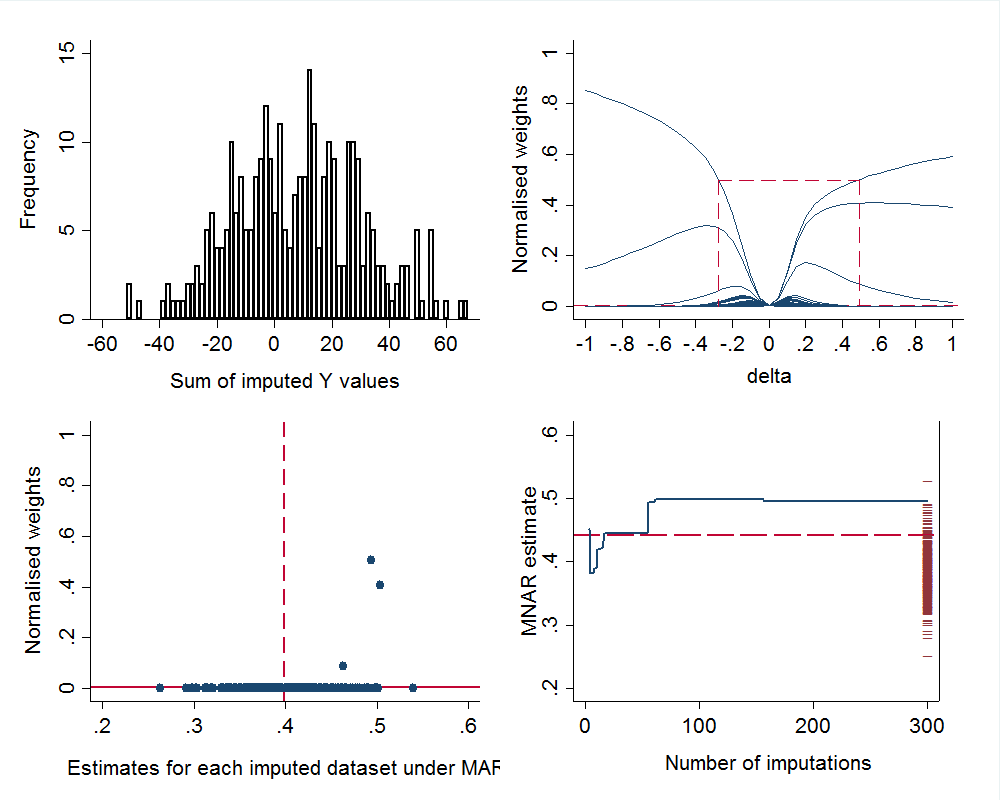

Supplement: Additional file 7: Figure S5. — Graphical procedure for selecting a range for δ (top). Top left panel: histogram of the sum of imputed Y values for each data set. Top right panel: graphical determination of delta for the variable Y using the approach suggested by Héraud-Bousquet et al. Graphical diagnostics (bottom). Bottom left panel: normalised weights against the MAR estimates obtained from each of the 500 imputed dataset. Bottom right panel: mean of the MNAR estimate against the number of imputations (n = 500, m = 300, δ (True) = 0.5, β (True) = 0.5, ${\widehat{\beta}}_{(Simulate)}$β^(Simulate) =0.501). (DOCX 2362 kb) [file 12874_2015_74_MOESM7_ESM.docx]

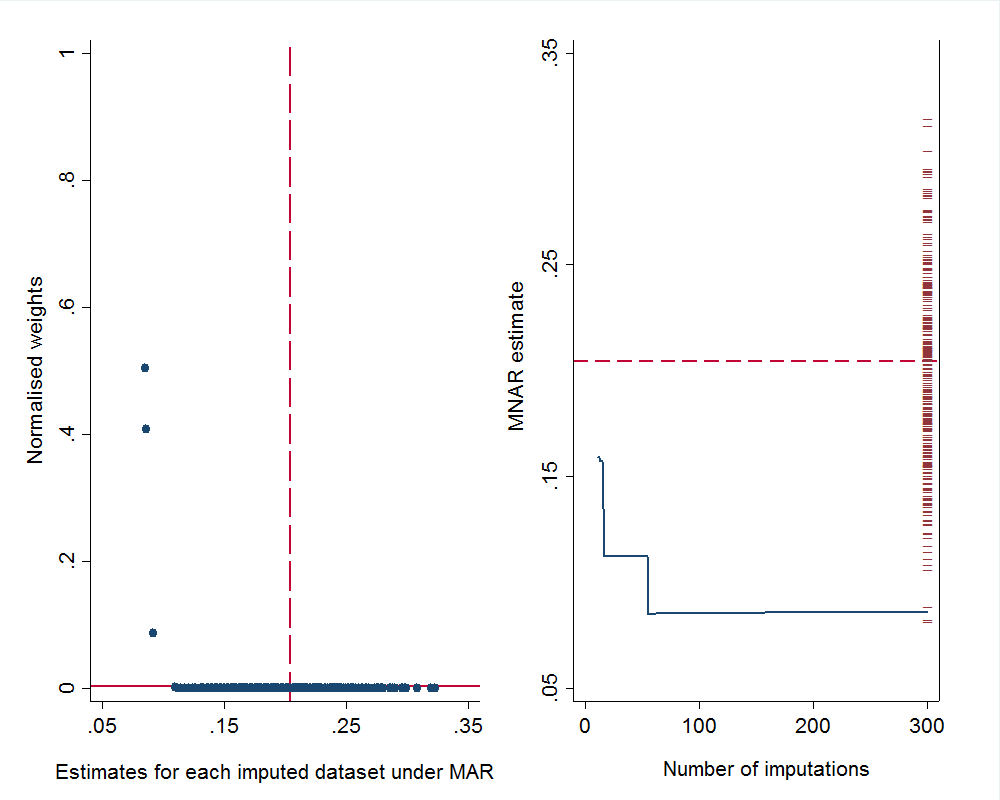

Supplement: Additional file 8: Figure S6. — Graphical diagnostics for a single simulated dataset (n = 500, m = 300, δ (True) =0.5, μ (True) =0, ${\widehat{\mu}}_{(Simulate)}$μ^Simulate = − 0.007). (DOCX 2362 kb) [file 12874_2015_74_MOESM8_ESM.docx]

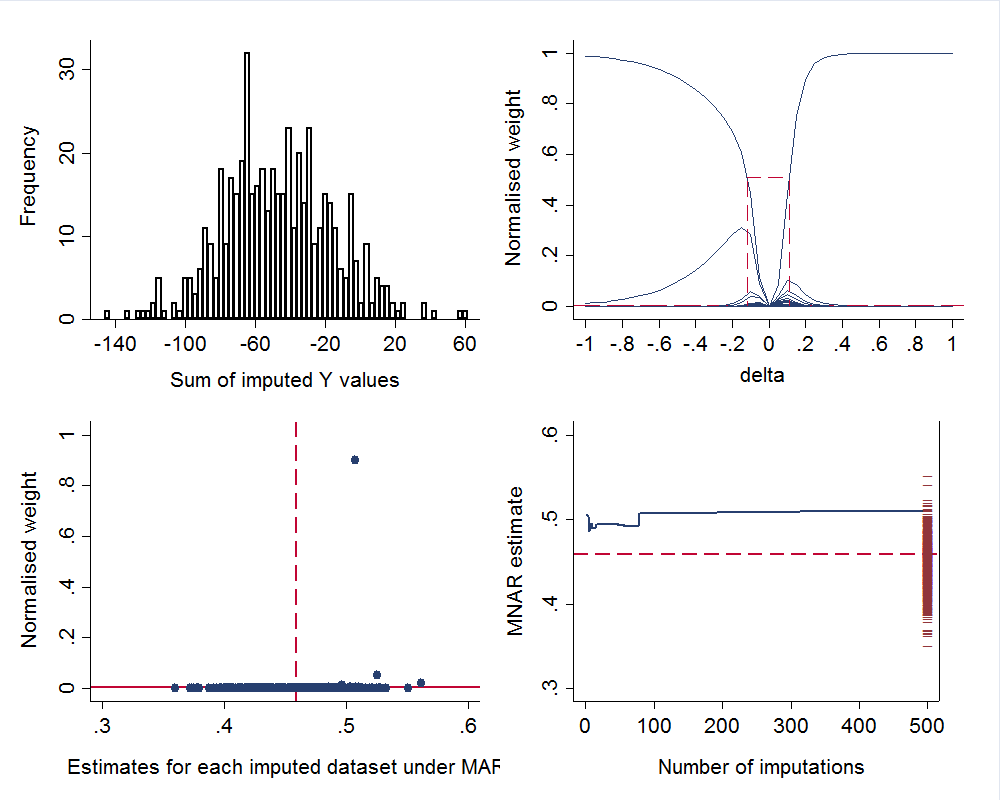

Supplement: Additional file 10: Figure S7. — Graphical procedure for selecting a range for δ (top). Top left panel: histogram of the sum of imputed Y values for each data set. Top right panel: graphical determination of delta for the variable Y using the approach suggested by Héraud-Bousquet et al. Graphical diagnostics (bottom). Bottom left panel: normalised weights against the MAR estimates obtained from each of the 500 imputed dataset. Bottom right panel: mean of the MNAR estimate against the number of imputations (n = 1000, m = 500, δ (True) = 0.2, β (True) = 0.5, ${\widehat{\beta}}_{(Simulate)}$β^Simulate =0.498). (DOCX 2362 kb) [file 12874_2015_74_MOESM10_ESM.docx]

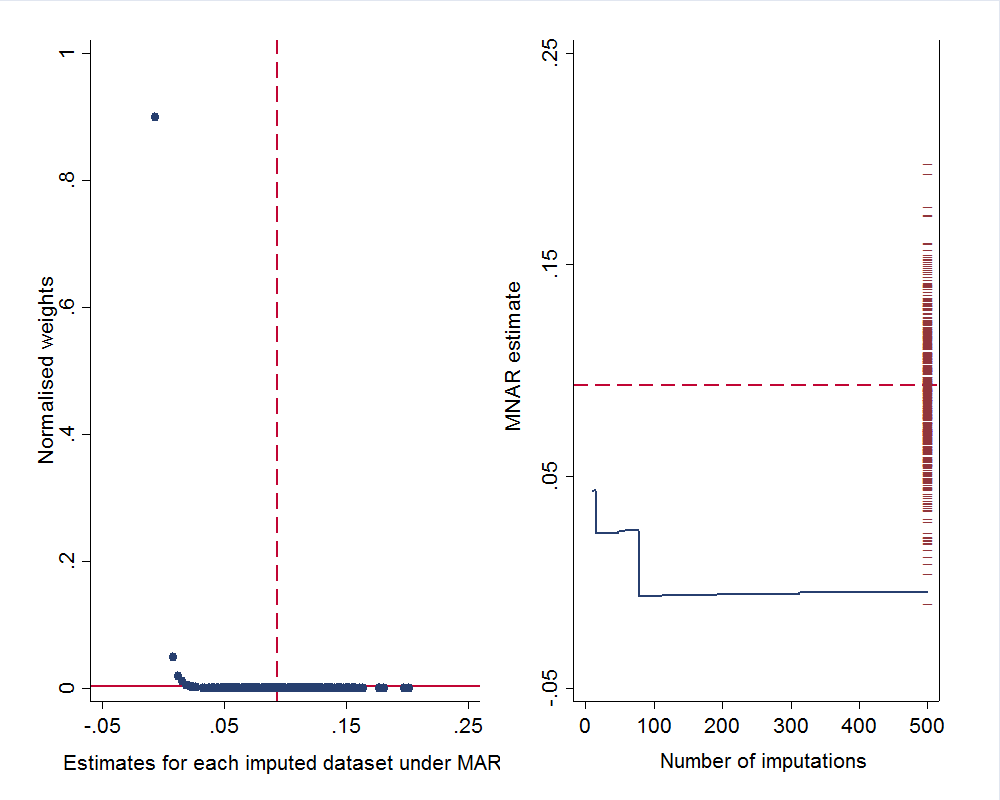

Supplement: Additional file 11: Figure S8. — Graphical diagnostics for a single simulated dataset (n = 1000, m = 500, δ (True) = 0.2, μ (True) = 0, ${\widehat{\boldsymbol{\mu}}}_{\left(\boldsymbol{Simulate}\right)}$μ^(Simulate) = − 0.009). (DOCX 2362 kb) [file 12874_2015_74_MOESM11_ESM.docx]
