# Supplementary material for: Evaluation of a weighting approach for performing sensitivity analysis after multiple imputation
Source: BMC Med Res Methodol. 2015 Oct 13;15:83. doi: 10.1186/s12874-015-0074-2 (PMC4604630; doi:10.1186/s12874-015-0074-2)
Supplement: Additional file 6: Table S2. — Estimates of the marginal mean of the normally distributed outcome variable and the regression coefficient under four analysis methods for a single simulated dataset (n = 500, m = 300, δ = 0.5). (DOCX 17 kb) [file 12874_2015_74_MOESM6_ESM.docx]

**Table S2 Estimates of the marginal mean of the normally distributed outcome variable and the regression coefficient under four analysis methods for a single simulated dataset *(n*=500*, m=*300*, δ*=0.5).**

|  | *μ* |  |  | *β* |  |
| --- | --- | --- | --- | --- | --- |
|  | Parameter estimate | SE |  | Parameter estimate | SE |
|  |  |  |  |  |  |
| Full dataset (before deletion) | -0.007 | 0.044 |  | 0.501 | 0.039 |
| Complete Case Analysis | 0.373 | 0.055 |  | 0.399 | 0.057 |
| Multiple Imputation under MAR | 0.204 | 0.041 |  | 0.398 | 0.058 |
| Sensitivity analysis under MNAR | 0.086 | 0.045 |  | 0.495 | 0.038 |
