# Supplementary material for: Evaluation of a weighting approach for performing sensitivity analysis after multiple imputation
Source: BMC Med Res Methodol. 2015 Oct 13;15:83. doi: 10.1186/s12874-015-0074-2 (PMC4604630; doi:10.1186/s12874-015-0074-2)
Supplement: Additional file 9: Table S3. — Estimates of the marginal mean of the normally distributed outcome variable and the regression coefficient under four analysis methods for a single simulated dataset (n = 1000, m = 500, δ = 0.2). (DOCX 17 kb) [file 12874_2015_74_MOESM9_ESM.docx]

**Table S3 Estimates of the marginal mean of the normally distributed outcome variable and the regression coefficient under four analysis methods for a single simulated dataset *(n*=1000*, m=*500*, δ*=0.2).**

|  | *μ* |  |  | *β* |  |
| --- | --- | --- | --- | --- | --- |
|  | Parameter estimate | SE |  | Parameter estimate | SE |
|  |  |  |  |  |  |
| Full dataset (before deletion) | -0.009 | 0.001 |  | 0.498 | 0.028 |
| Complete Case Analysis | 0.282 | 0.002 |  | 0.459 | 0.043 |
| Multiple Imputation under MAR | 0.093 | 0.044 |  | 0.459 | 0.043 |
| Sensitivity analysis under MNAR | -0.005 | 0.032 |  | 0.509 | 0.028 |
